# Species of *Elasmogorgia* and *Euplexaura* (Cnidaria, Octocorallia) from Japan with a discussion about the genus *Filigella*

**DOI:** 10.3897/zookeys.589.8361

**Published:** 2016-05-16

**Authors:** Asako K. Matsumoto, Leen P. van Ofwegen

**Affiliations:** 1 Planetary Exploration Research Center (PERC), Chiba Institute of Technology (Chitech), Tsudanuma 2-17-1, Narashino, Chiba 275-0016, Japan; 2 Naturalis Biodiversity Center, Darwinweg 2, P.O. Box 9517, 2300 RA Leiden, The Netherlands

**Keywords:** Astrogorgia, Thesea, Plexauridae, Alcyonacea, deep-water octocorals, Indo-Pacific, new species, Challenger Expedition

## Abstract

Octocorals with thread-like colony shape have been re-examined, mainly from Japanese waters. The holotypes of *Elasmogorgia filiformis* and *Filigella boninensis* and a syntype of *Filigella mitsukurii* have been studied. *Euplexaura arbuscula* is identified and *Euplexaura yayoii* **sp. n.** described.

## Introduction

The octocoral genera *Elasmogorgia*, *Filigella* and *Thesea* have been underexplored and their taxonomic position remains confusing. One of the Japanese species of these genera, *Filigella mitsukurii*, is classified with three different genera in WoRMS, as *Elasmogorgia mitsukurii* (Ofwegen 2016a), *Filigella mitsukurii* (Ofwegen 2016b), and *Thesea mitsukurii* (Ofwegen 2016c). In this manuscript, a revision is presented of the genera *Elasmogorgia* and *Filigella* and their species in Japan, as well as some Japanese species of *Euplexaura*.

The genus *Filigella* Gray, 1868 was established to accommodate *Filigella gracilis* from Brazil. Later on Wright and Studer (1899) established the Pacific genus *Elasmogorgia* with the remark that their new species *Elasmogorgia filiformis* could be identical to *Filigella gracilis*. Next, Hickson (1905) described *Elasmogorgia flexilis* from the Maldives, Kinoshita (1909) described *Filigella mitsukurii* from Japan, Nutting (1912) described *Elasmogorgia ramosa*, also from Japan, and finally Aurivillius (1931) described *Filigella boninensis* from the Ogasawara Islands (Bonin Islands), and Thomson and Dean (1931) described *Elasmogorgia filigella* from Kalimantan (Indonesia). Both Kinoshita and Aurivillius considered *Elasmogorgia* and *Filigella* synonymous and Aurivillius doubted whether *Elasmogorgia ramosa* of Nutting (1912) belonged to one of these two genera. Kükenthal (1919) first treated them as two separate genera but he synonymized them five years later (Kükenthal 1924).

Bayer (1959: 17) was the first to include *Filigella gracilis* in the genus *Thesea* Duchassaing & Michelotti, 1860, although he did not directly synonymize the genus *Filigella* with *Thesea*, but much later in his key to the octocoral genera, Bayer (1981: 945). However, he did not re-examine six Pacific species referred to *Filigella* or *Elasmogorgia*, and therefore the status of these species has remained doubtful.

In the present study, the type material of *Elasmogorgia filiformis*, *Elasmogorgia filigella*, *Filigella mitsukurii*, and *Filigella boninensis*, is examined and their previous identifications are discussed. In addition, two specimens identified as *Elasmogorgia filiformis* by Nutting (1910) and by Thomson and Dean (1931) were examined. *Elasmogorgia filigella* Thomson and Dean (1931) from Kalimantan clearly does not belong to *Elasmogorgia* because it has a red colony and also red sclerites. The type specimen of *Elasmogorgia filigella* (ZMA 2536) appears to consist of a few branch fragments with disintegrated sclerites. It is considered to represent a species of *Astrogorgia* in the present study.

**Figure 1.**
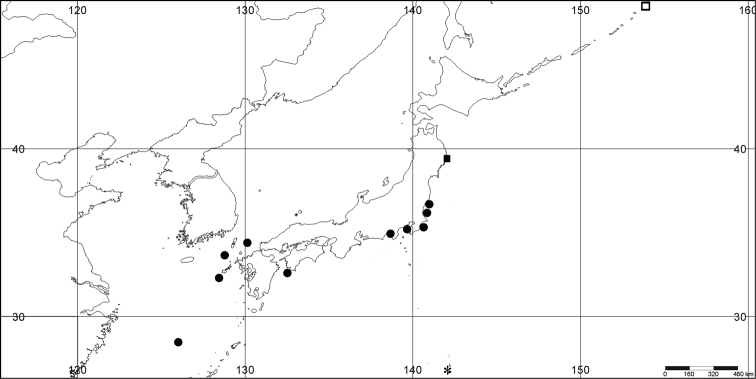
Distribution map of *Euplexaura boninensis* (*), *Elasmogorgia mitsukurii* (●), *Euplexaura arbuscula* (□), and *Euplexaura yayoii* sp. n. (■).

Finally, a new thread-like *Euplexaura* species is decribed from the Pacific side of northern Japan, *Euplexaura yayoii* sp. n., in addition to *Euplexaura arbuscula* Broch, 1935 from off Chishima Is. (Kuril Is.), which previously was reported from the west coast of Kamchatka, Sea of Okhotsk. These two species are both from northern Japan and northeastern Russia (Figure 1).

## Material and methods

### Abbreviations

BMNH
 British Museum of Natural History, London, UK 


NBC (RMNH)
Naturalis Biodiversity Center, formerly Rijksmuseum van Natuurlijke Historie, Leiden, The Netherlands 


UMUTZ
University Museum of University of Tokyo, Tokyo, Japan 


UUZM (UPSZTY)
Museum of Evolution, Uppsala, Sweden 


ZMUC
Zoological Museum University of Copenhagen, Copenhagen, Denmark 


ZIN
Museum of the Zoological Institute of the Russian Academy of Sciences St. Petersburg, Russia 


ZMA
Zoological Museum Amsterdam (ZMA), now part of NBC 


### Material

Material was collected from depths between 38 and 366 m by dredging, trawling or fishing net onboard *RV Tansei-maru*, University of Tokyo and Japan Agency for Marine-earth Science and Technology and *RV Yayoi*, the University of Tokyo, during the years 1975–2010. Type specimens and other historical museum material was examined in collections of the BMNH, NBC, UMUTZ, UUZM, ZIN, and ZMUC.

From each specimen a small piece of the distal part of a branch was dissolved in a solution of household bleach (4% hypochlorite) to isolate sclerites. The sclerites were washed with demineralised water, dried on a hot plate, mounted on SEM stubs, and coated with Pd/Au for SEM imaging. For this, either a JEOL JSM6490LV scanning electron microscope was operated at high vacuum at 10 kV, or a JEOL JSM6510LA scanning electron microscope with a Quick Carbon Coater SC-701C, SANYU ELECTRON was used. For terminology, see Bayer et al. (1983).

Descriptions of old Japanese material collected by Japanese used “hiro” (Japanese fathom) as the depth unit. One Japanese fathom (hiro) is usually 1.43 m, occasionally 1.51 m, whereas, it is 1.818 m for the length unit on land. The old depth unit fathom is also converted to 1.8288 m. When it was not clear whether the collector used fathom or hiro, the converted depth has wider ranges.

## Taxonomy

### Genus Elasmogorgia

Taxon classificationAnimaliaAlcyonaceaPlexauridae

Wright & Studer, 1889

Elasma (non Elasma Jaennicke 1866); Studer (and P. Wright) 1887: 58.
Elasmogorgia Wright & Studer, 1899: 132; Kükenthal 1919: 836; Soest 1979: 88.
Elasmogorgia ? Elasmogorgia; Hickson 1905: 814; Thomson and Simpson 1909: 238; Thomson and Russell 1910: 159; Nutting 1912: 85.
Elasmogorgia NOT Elasmogorgia; Nutting 1909: 717; 1910: 45; 1912: 85; Thomson and Dean 1931: 199.
Elasmogorgia Partly Elasmogorgia; Kükenthal 1924: 148.

#### Diagnosis.

Plexauridae with sparsely branched colonies lacking a holdfast. Calyces dome-shaped. Polyps with collaret and points. Sclerites are colourless spindles.

### Elasmogorgia filiformis

Taxon classificationAnimaliaAlcyonaceaPlexauridae

Wright & Studer, 1889

Figures 2a , 3 , 4

Elasmogorgia filiformis Wright & Studer, 1889: 133 (Indonesia, Arafura Sea); Kükenthal 1924: 148.
Elasmogorgia filiformis ? Elasmogorgia filiformis; Nutting 1912: 85 (Tateisha zaki Light, Japan); Thomson and Russell 1910: 159 (Amirantes); Thomson and Simpson 1909: 238 (Birma, India); Tixier-Durivault 1966: 403 (Madagascar); all not re-examined.
Elasmogorgia filiformis NOT Elasmogorgia filiformis; Nutting 1909: 717 (California = Thesea); 1910: 45 (Timor = Euplexaura); Thomson and Dean 1931: 199 (Sulawesi = Astrogorgia).

#### Material examined.

Holotype BMNH 1889.5.27.77, Arafura Sea, South of Papua, 28 fms, Challenger st. 188, 10 September 1874; ZMA Coel. 2537, Siboga st. 213, Saleyer anchorage, Sulawesi, Indonesia, 38 m, 26 September 1899 (= *Astrogorgia*); ZMA Coel. 2538, Timor, 112 m, Siboga st. 289, 09°00.3'S, 126°24.5'E (= *Euplexaura*).

#### Diagnosis.

**Figure 2.**
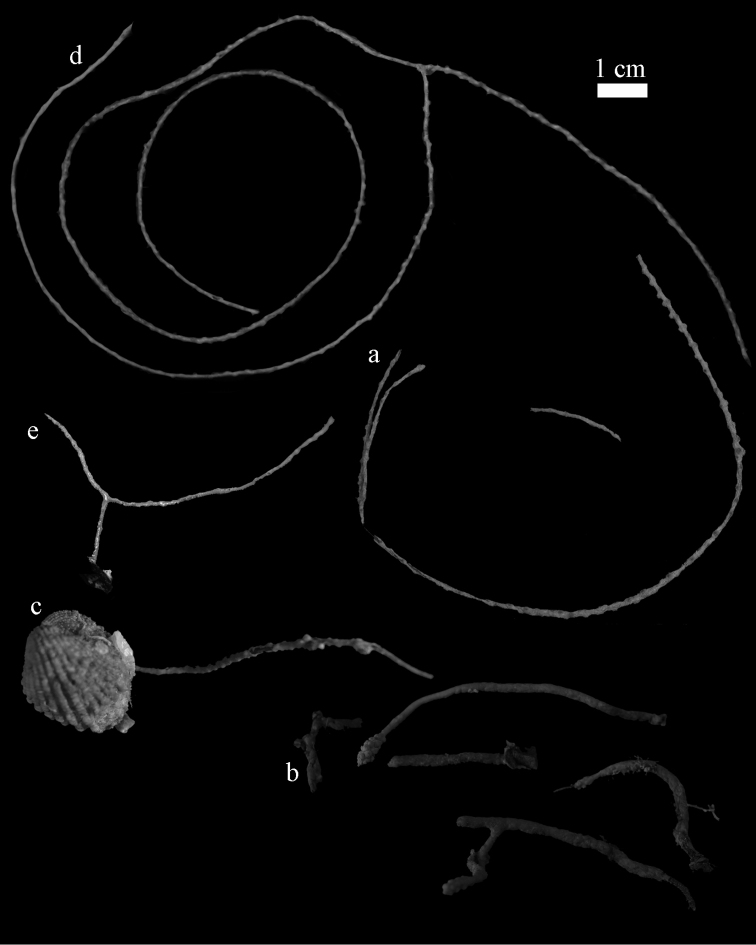
**a** *Elasmogorgia filiformis* Wright & Studer, 1889, holotype BMNH 1889.5.27.77 **b** *Euplexaura arbuscula* Broch, 1935, ZIN 11667 **c** *Euplexaura boninensis* (Aurivillius, 1931), holotype UPSZTY2165 (UUZM 68) **d** *Elasmogorgia mitsukurii* (Kinoshita, 1909), syntype UMUTZ-CnidG-222 **e** *Euplexaura yayoii* sp. n., holotype RMNH 42104.

**Figure 3.**
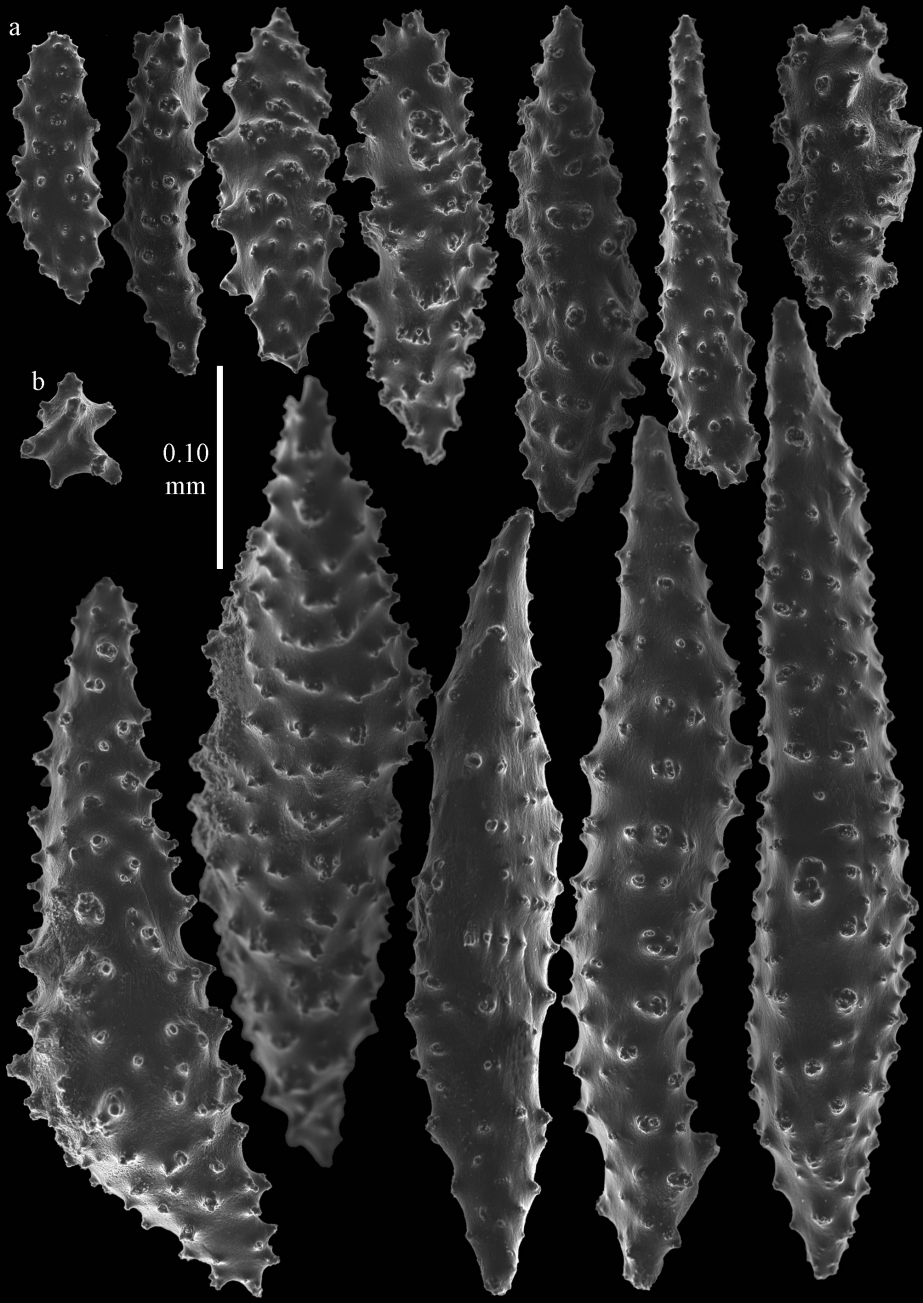
*Elasmogorgia filiformis* Wright & Studer, 1889, holotype BMNH 1889.5.27.77, **a** spindles from surface layer of coenenchyme **b** capstan.

**Figure 4.**
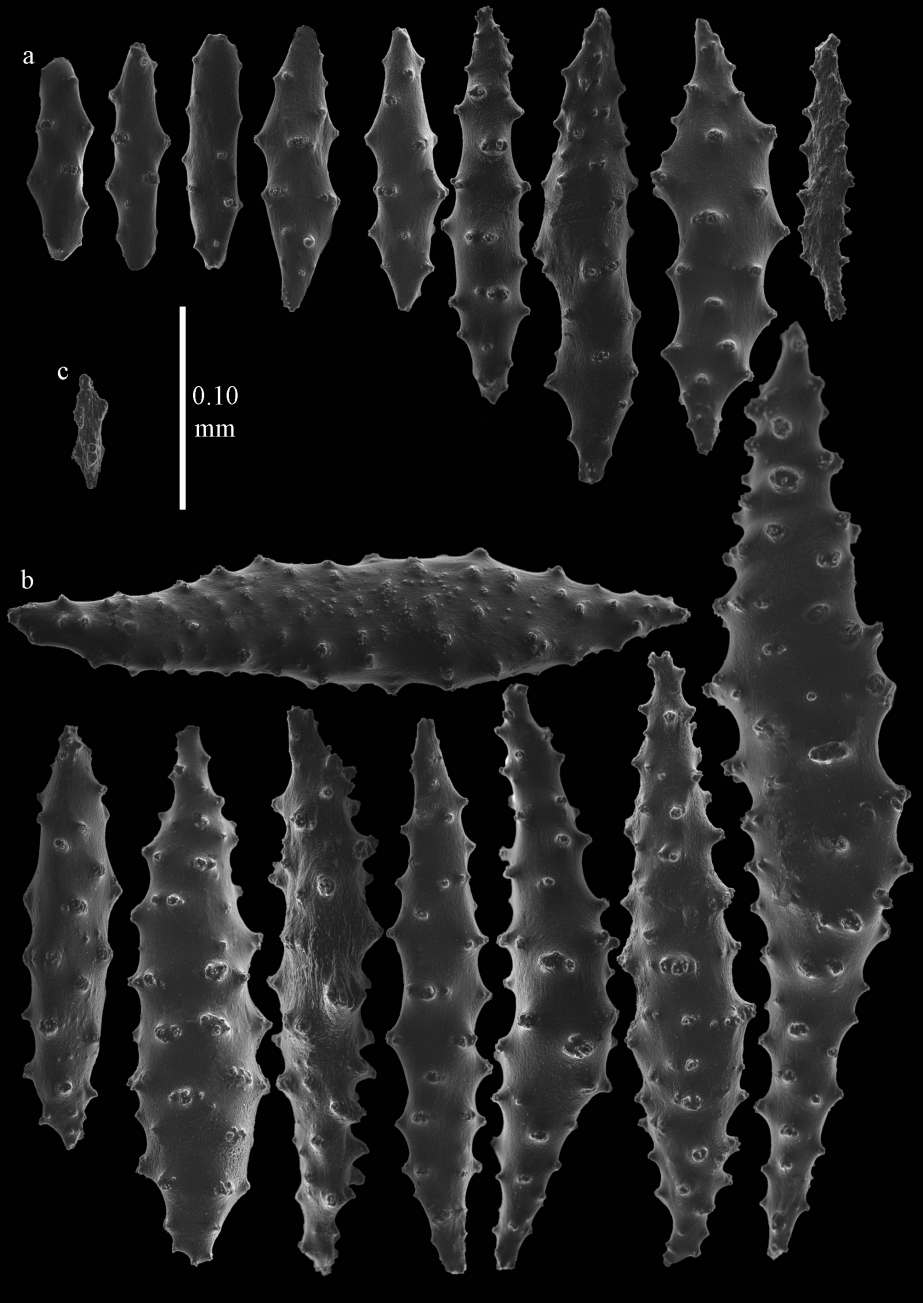
*Elasmogorgia filiformis* Wright & Studer, 1889, holotype BMNH 1889.5.27.77, **a** spindles from interior of coenenchyme **b** possible collaret spindle **c** rod.

Colony thread-like (Figure 2a). Calyces dome-shaped, arranged all around the branches (Figure 2a). Coenenchyme with spindles up to 0.45 mm long, with simple tubercles (Figures 3–4). Colony white with colourless spindles.

#### Remarks.

One somewhat flattened spindle was found, 0.35 mm long, maybe referable to a collaret (Figure 4b), and one capstan (Figure 3b). As the microscope slide that was made only shows heavily oxidized black sclerites it could not be really ascertained where different types of sclerites came from. The little fragment available was not sufficient for more extensive examination. Wright and Studer (1889) mentioned spindles up to 0.62 mm long. They also mentioned the basal portion of the tentacles has spindle-shaped sclerites of up to 0.18 mm long. *Elasmogorgia filiformis* mostly resembles a species of *Astrogorgia* but differs in not having polyp body sclerites and extremely weak ornamentation of spindles. Until new material becomes available for a more thorough examination the genus *Elasmogorgia* is retained.

ZMA 2537 of Thomson and Dean (1931) is a thread-like colony fragment containing colourless disintegrated sclerites, which were sufficiently recognizable to identify it as a species of *Astrogorgia*. In a comparison with *Astrogorgia bayeri* Ofwegen and Hoeksema, 2001, from Sulawesi, the latter species appears to have shorter spindles, up to 0.5 mm long, whereas Thomson and Dean's (1931) specimen has spindles of over 1 mm long. Because of the disintegrated state of its sclerites, no more differences could be ascertained.

ZMA 2538 of Nutting (1910) was also re-examined; it has characters of the genus *Euplexaura*. *Elasmogorgia filiformis* of Nutting (1912) is also unlikely an *Elasmogorgia*.

### Genus Euplexaura

Taxon classificationAnimaliaAlcyonaceaPlexauridae

Verrill, 1869

Euplexaura Verrill, 1869: 75; Kükenthal 1924: 90 (synonymy of the genus).

#### Diagnosis.

Plexauridae with colonies branched in one plane. Calyces may be present but are mostly absent. Polyps with collaret and points, only point sclerites, or no sclerites at all. The surface of the coenenchyme with robust ovals or spindles with complex tubercles; sometimes with one side that is less tuberculate. The interior with rods or small spindles with simple tubercles. All sclerites colourless.

### Euplexaura arbuscula

Taxon classificationAnimaliaAlcyonaceaPlexauridae

Broch, 1935

Figures 1 , 2b , 5

Euplexaura arbuscula Broch, 1935: 20, fig. 12.

#### Material.

ZIN 11667(ZIN110824-018-040), Skaly Lovushki I., off Chishima Is. (= Kuril Is.), 154°44'5E, 48°15'5N, depth 140 m, Bottom: gravel with stones, Ship *Odissey*, Grab “Ocean” 50 cm^2^ (bottom sampler), coll. Boris Sirenko and Mikhail Kolesnikov, 3 August 1984.

#### Diagnosis.

**Figure 5.**
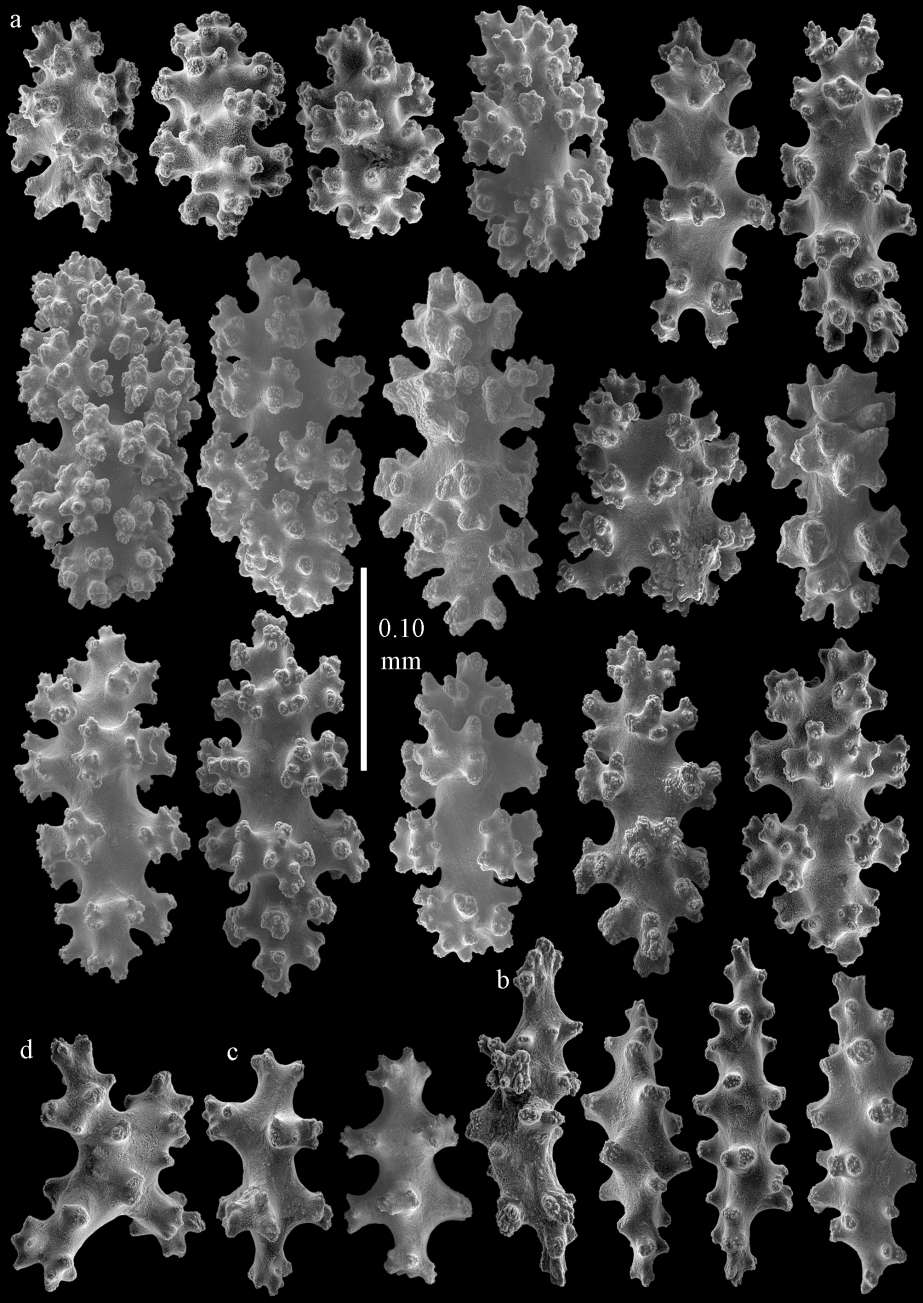
*Euplexaura arbuscula* Broch, 1935, ZIN 11667 **a** spindles and blunt ellipsoids from surface layer of coenenchyme **b–d** sclerites from interior of coenenchyme **b** spindles **c** capstans **d** cross.

Branches thread-like. Calyces dome-shaped, arranged all around the branches (Figure 2b). Polyps without sclerites. The surface layer of the coenenchyme has spindles and blunt ellipsoids (Figure 5a), up to 0.15 mm long, with complex tubercles. The interior has small spindles, capstans, and a few crosses, up to 0.15 mm long (Figures 5b-d), all with simple tubercles.

#### Remarks.

The material examined was fragmentary (Figure 2b) and therefore it resembles a species of *Elasmogorgia*.

*Euplexaura abietina* Kukenthal, 1909 resembles *Euplexaura arbuscula* regarding its sclerites, but it differs in having polyp spindles.

Since its original description, the species was not found again and its type material could not be retraced, hence some doubts remain about the identification of this species. Broch (1935) described the species only from one specimen. It is not present in the Natural History Museum, University of Oslo, and ZIN.

#### Distribution.

Kamchatka, Sea of Okhotsk, off Chishima Is. (= Kuril Is.).

### Euplexaura boninensis

Taxon classificationAnimaliaAlcyonaceaPlexauridae

(Aurivillius, 1931)

**Figure 6.**
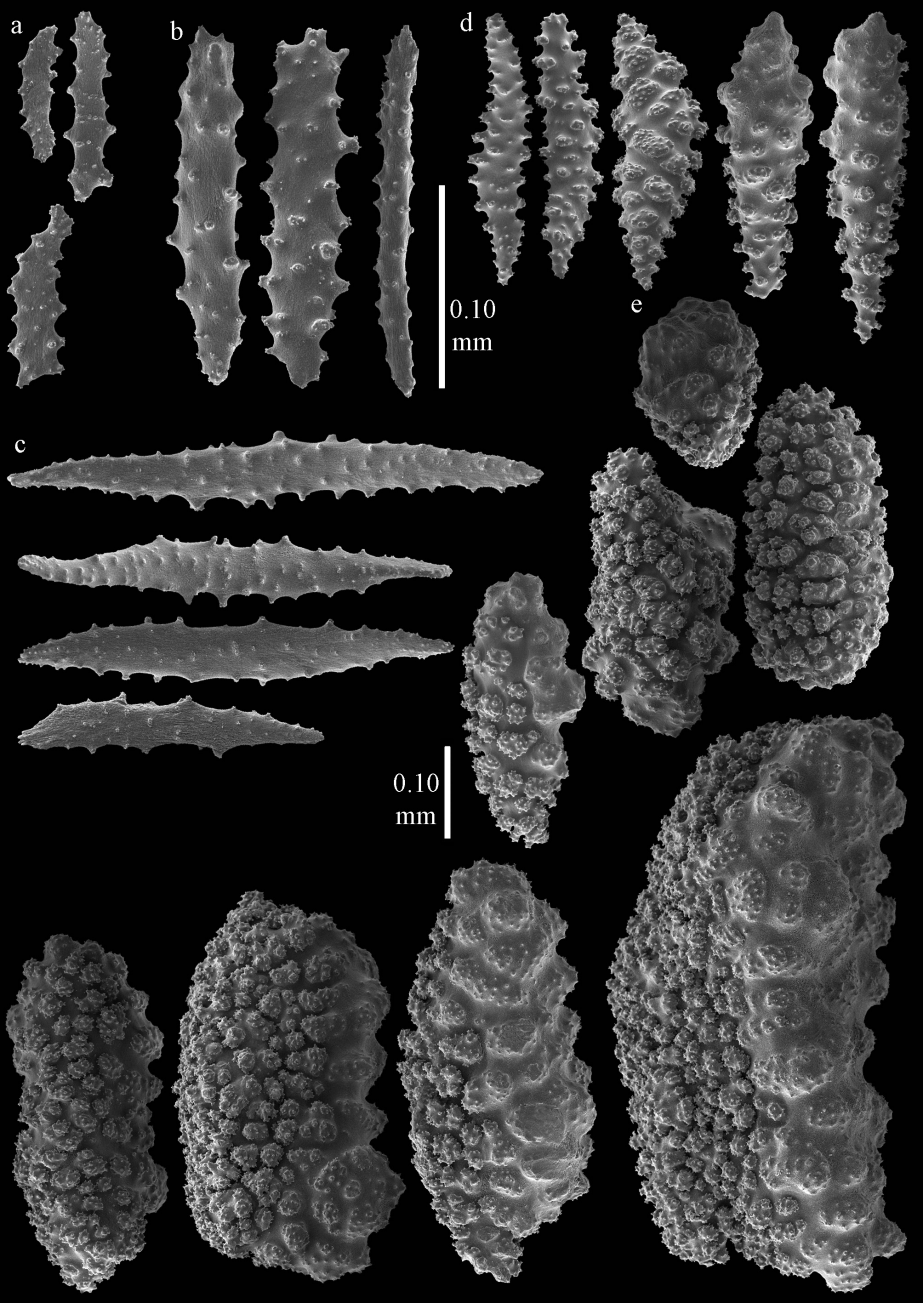
*Euplexaura boninensis* (Aurivillius, 1931), holotype UPSZTY2165 (UUZM 68), **a** tentacle scales **b** point spindles **c** collaret spindles **d** spindles from surface layer of coenenchyme **e** blunt ellipsoids from surface layer of coenenchyme.

**Figure 7.**
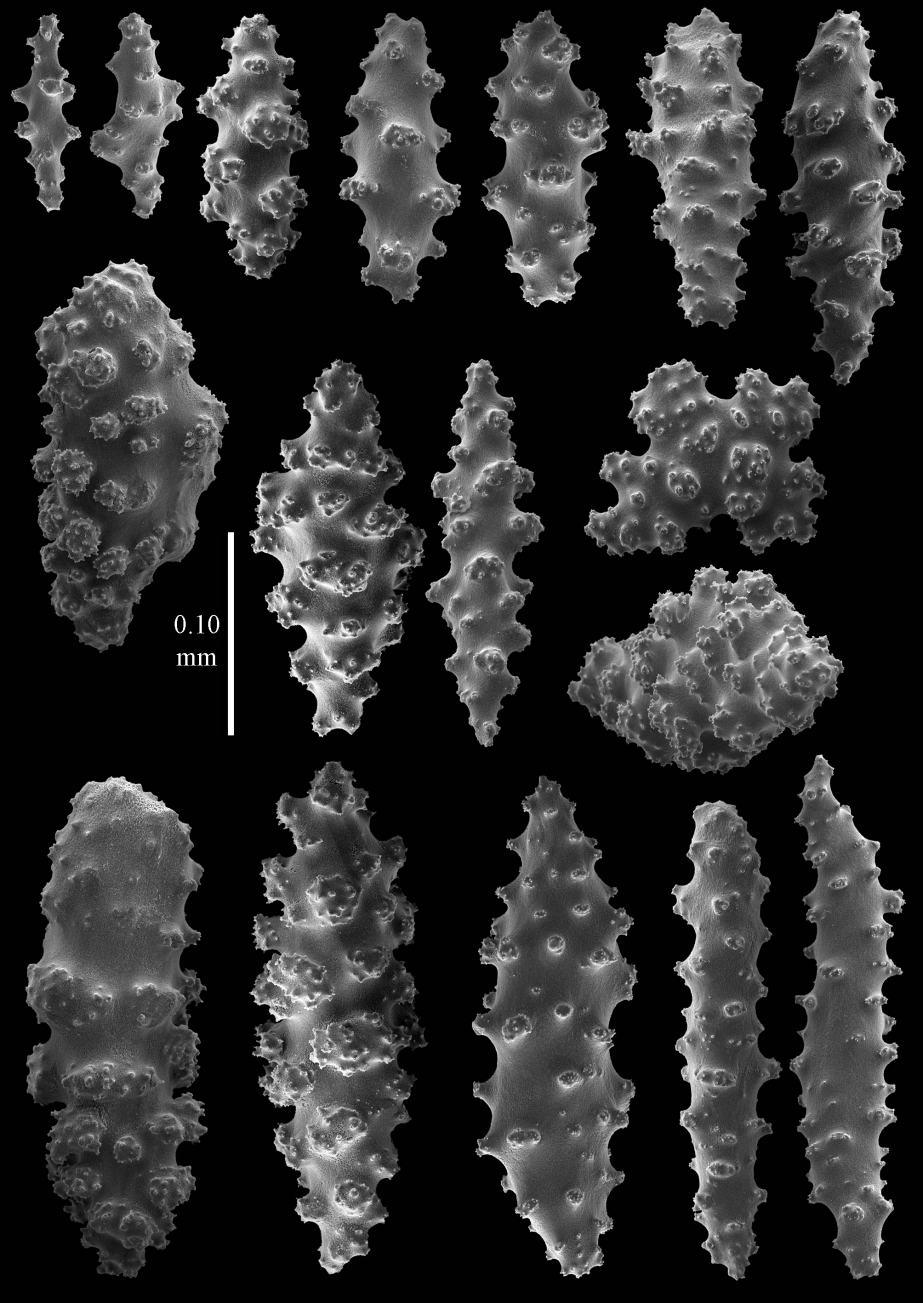
*Euplexaura boninensis* (Aurivillius, 1931), holotype UPSZTY2165 (UUZM 68), sclerites of interior of coenenchyme.

Figures 1 , 2c , 6 , 7

Filigella boninensis Aurivillius, 1931: 139 (Bonin Islands).
Thesea boninensis ; Matsumoto 2014: 158 (Table 1, listed only).

#### Material examined.

Holotype UPSZTY2165 (UUZM 68), East of Chichijima I., Ogasawara Is. (= Bonin Is.), Japan, depth 100 m, in formalin, Dr. Sixten Bock's, Japan Expedition, coll. Dr. Sixten Bock, 1 August 1914.

#### Diagnosis.

Branches thread-like, 6 cm in length. Calyces dome-shaped, arranged all around the branches (Figure 2c). The polyps have points with flattened spindles, up to 0.15 mm long (Figure 6b), with simple tubercles and spiny distal end. Collaret present, with slightly bent, flattened spindles, up to 0.25 mm long, with simple tubercles (Figure 6c). Tentacles with small scales, up to 0.10 mm long (Figure 6a).

The surface layer of the coenenchyme has spindles (Figure 6d) and blunt ellipsoids (Figure 6e), up to 0.65 mm long, with complex tubercles. Several of them with one side less tuberculate. The interior has small spindles and rods, up to 0.25 mm long (Figure 7), with simple tubercles.

#### Remarks.

Because the sclerites of this species are spindles and ellipsoids with complex tubercles it actually represents an *Euplexaura* species. It is the only species of *Euplexaura* with thread-like colony shape which has many sclerites with one side that is less tuberculate.

### Euplexaura mitsukurii

Taxon classificationAnimaliaAlcyonaceaPlexauridae

(Kinoshita, 1909)

**Figure 8.**
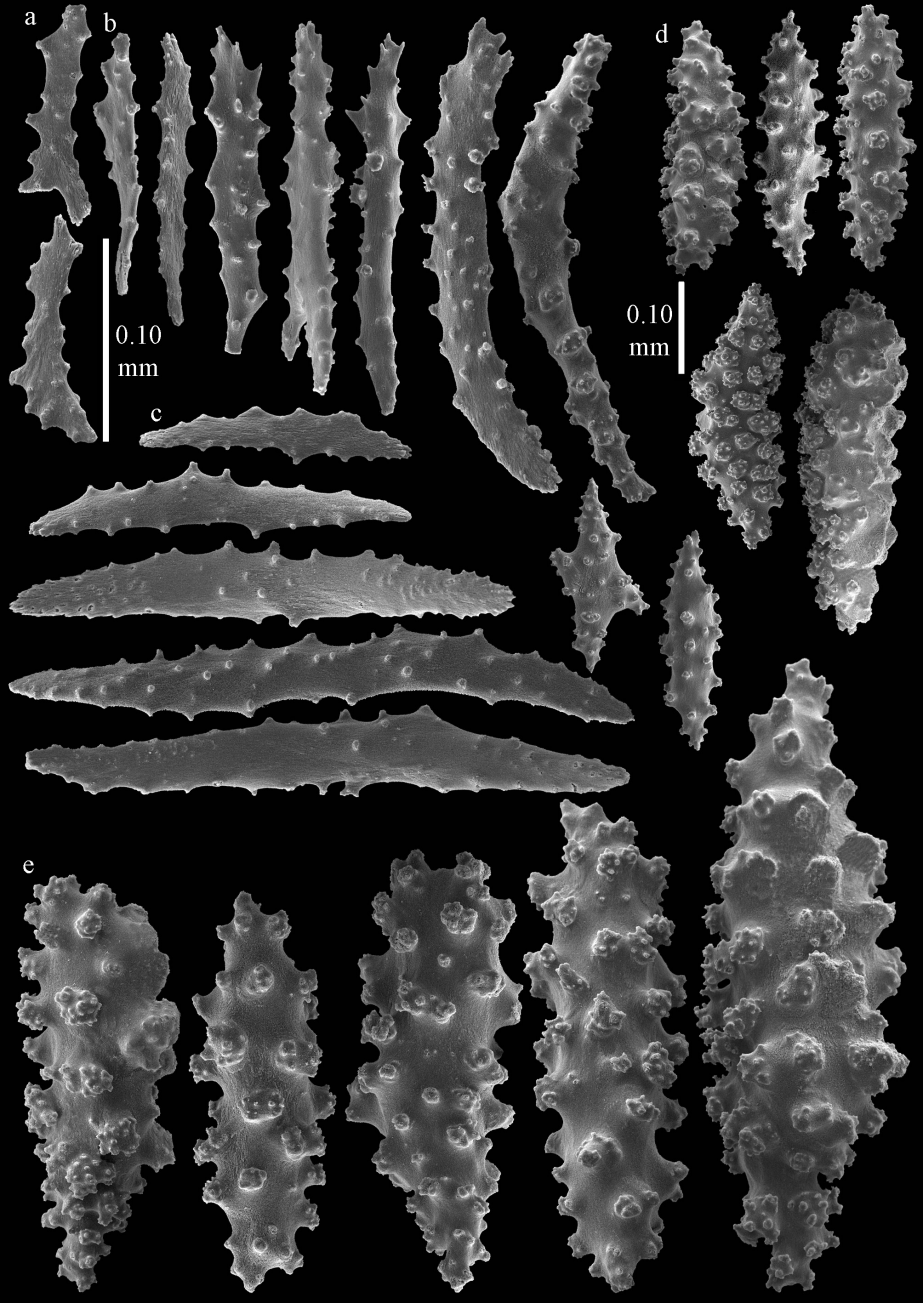
*Euplexaura mitsukurii* (Kinoshita, 1909), syntype UMUTZ-CnidG-222 **a** tentacle scales **b** point spindles **c** collaret spindles **d–e** spindles from surface layer of coenenchyme. Scale at d only applies to **d**.

**Figure 9.**
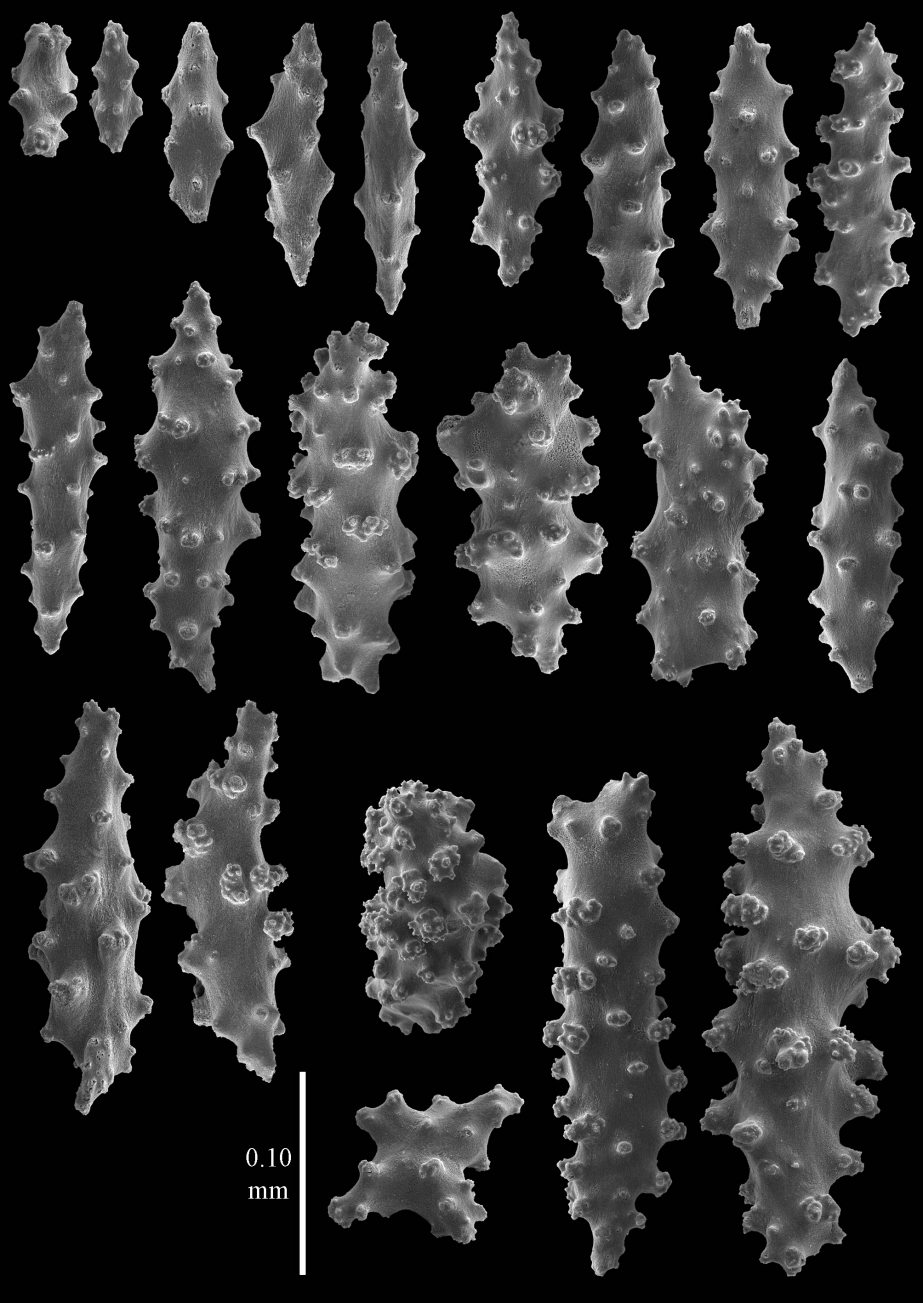
*Euplexaura mitsukurii* (Kinoshita, 1909), syntype UMUTZ-CnidG-222 sclerites of interior of coenenchyme.

Figures 1 , 2d , 8 , 9

Filigella mitsukurii Kinoshita, 1909: 1(Sagami Bay); Aurivillius 1931: 129 (Kiu-Shiu = Kyushu); Utinomi 1961: 213; Bayer 1956: F206, fig. 148,3.
Elasmogorgia mitsukurii ; Kükenthal 1924: 149.
Thesea mitsukurii ; Matsumoto et al. 2007: 240 (Table 2, listed); Matsumoto 2014: 158, 159, 160 (Table 1, listed).
Thesea sp. Matsumoto 2014: 161 (Table 1, listed).

#### Material examined.

Syntypes UMUTZ-CnidG-222, off Jogashima I., Sagami Bay, Japan, depth 70 Japanese fathoms (100-106 m), secured with Hydra dredge, 26 August 1901; UMUTZ-CnidG-223, Japanese 2 nautical miles (5 km in Kinoshita, 1909) of West South off Jogashima I., Sagami Bay, Japan, saba-nawa line, 31 July 1892. Identified museum material UMUTZ-CnidG-122, off Torishima I., Japan, East China Sea, 28°10'N, 126°2'E - 28°20'N, 126°11'E, depth 64 fms (117 m), trawl, coll. N. Yanaghi, 22 June 1913, det. F.M. Bayer, ca.1950, as *Filigella mitsukurii*; UMUTZ-CnidG-126 same data as UMUTZ-CnidG-122, as *Filigella mitsukurii*. Unidentified museum material. ZMUC ANT-000611 (ZMUC120604-09), East China Sea, 33°41'N, 128°50'E, depth 75 fms (137 m), sand, *Hyateri maru*, trawl, coll. Dr. Th. Mortensen, 17 May 1914; ZMUC ANT-000616 (ZMUC120604-16), East China Sea, 32°15'N, 128°17'E, depth 90 fms (165 m), hard bottom, *Hyateri maru*, coll. Dr. Th. Mortensen, 15 May 1914; ZMUC ANT-000664 (ZMUC120604-59), 34°20'N, 130°10'E, depth 60 fms (110 m), sand, coll. Dr. Th. Mortensen, 18 May 1914; ZMUC ANT-000655 (ZMUC120604-67), off Misaki Biological Station, Sagami Bay, Japan, depth 200 fms (366 m), sand, coll. Dr. Th. Mortensen, 30 June 1914; AKM1630, Sukumo Bay, Bungo Channel, Japan, ca.32°38'N, ca.132°29’-30'E, depth 144-150 m, *RV Tansei-maru*, KT86-16, st.A-8, 1 m ORI biological dredge, coll. S. Ohta, 1 November 1986; AKM1631, off Kashima, Kashima Sea, Japan, 36°07'N, 140°49.0'E, depth 63-71 m, *RV Tansei-maru*, KT79-13, st. KB2, 2 m Beam trawl, coll. S. Ohta, 7 August 1979; AKM1632, off Toi, Suruga Bay, Japan, depth 192-207 m, 34°55.83'N, 138°44.85'E - 34°56.62'N, 138°45.0'E, *RV Tansei-maru*, KT75-15, st. 02, 2 m Beam Trawl, coll. S.Ohta, 24 November 1975; AKM1566, South East off Taito-saki Cape, Boso Peninsula, Japan, 35°21.259'N, 140°45.27'E - 35°21.359'N, 140°45.613'E, depth 104-105 m, *RV Tansei-maru*, KT01-08, st. TZ-7, 1 m ORI biological dredge, coll. S. Ohta, 22 June 2001; AKM 1644, off Hitachi, Kashima sea, Japan, 36°36.4'N, 140°50.1'E - 36°35.2'N, 140°50.5'E, depth 79–82 m, *RV Tansei-maru*, KT79-13, st. KB14, 2 m Beam trawl, coll. S. Ohta, 9 August 1979.

#### Diagnosis.

Branches thread-like. The examined syntype has two branches arising from the main stem with a length of 19 cm and 28.5 cm, respectively; the main stem is 9 cm long. Calyces dome-shaped, arranged all around the branches (Figure 2d). The polyps have points with flattened spindles, up to 0.20 mm long (Figure 8b), with simple tubercles. Collaret present, with slightly bent, flattened spindles, up to 0.30 mm long, with sparse, simple tubercles (Figure 8c). Tentacles with small scales, up to 0.10 mm long (Figure 8a).

The surface layer of the coenenchyme has spindles (Figure 8d–e), up to 0.35 mm long, with complex tubercles. Some of them with one side that is less tuberculate. The interior has small spindles and rods, up to 0.25 mm long (Figure 9), with simple tubercles.

#### Remarks.

Because the sclerites of this species are spindles with complex tubercles this is actually a species of *Euplexaura*.

Kinoshita (1909) mentioned 13 specimens of *Filigella mitsukurii* and three of them were complete. He used two specimens for his original description. Nowadays two specimens are present in UMUT and the data fit Kinoshita's, two specimens in his description.

The locality name “Jogaschima, Pagamibai” of this species in Kükenthal (1924) is a mistyping of “Jogashima, Sagamibai (Sagami Bay)”.

#### Distribution.

Sagami Bay, off Boso Peninsula, Kashima Sea, Suruga Bay, Bungo Channel, East China Sea, Japan.

### Euplexaura yayoii sp. n.

Taxon classificationAnimaliaAlcyonaceaPlexauridae

http://zoobank.org/65B660AC-70D9-4697-8411-A1F3116FFD47

**Figure 10.**
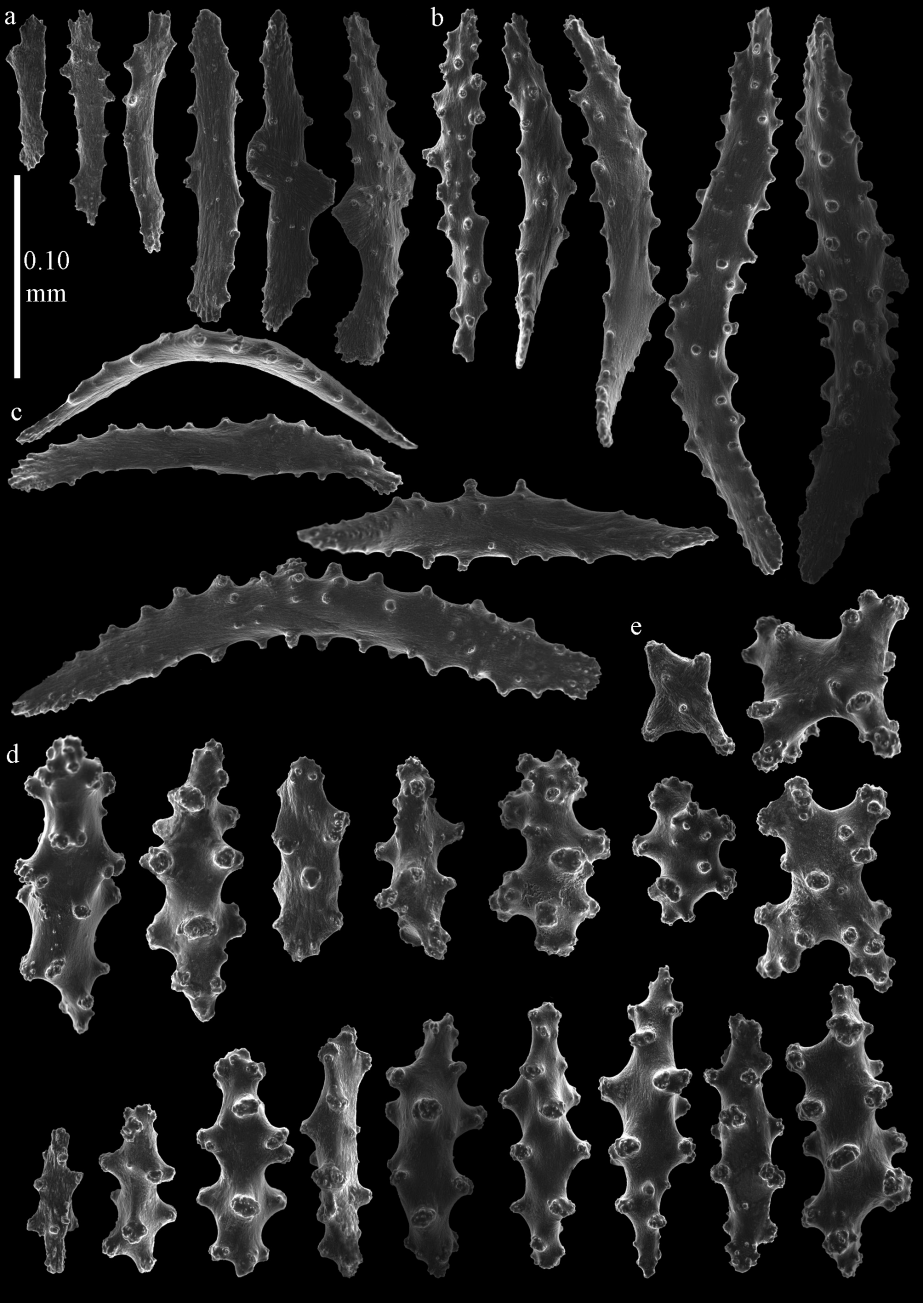
*Euplexaura yayoii* sp. n., holotype RMNH 42104 **a** tentacle scales **b** point spindles **c** collaret spindles **d–e** sclerites of interior of coenenchyme **d** spindles **e** crosses.

**Figure 11.**
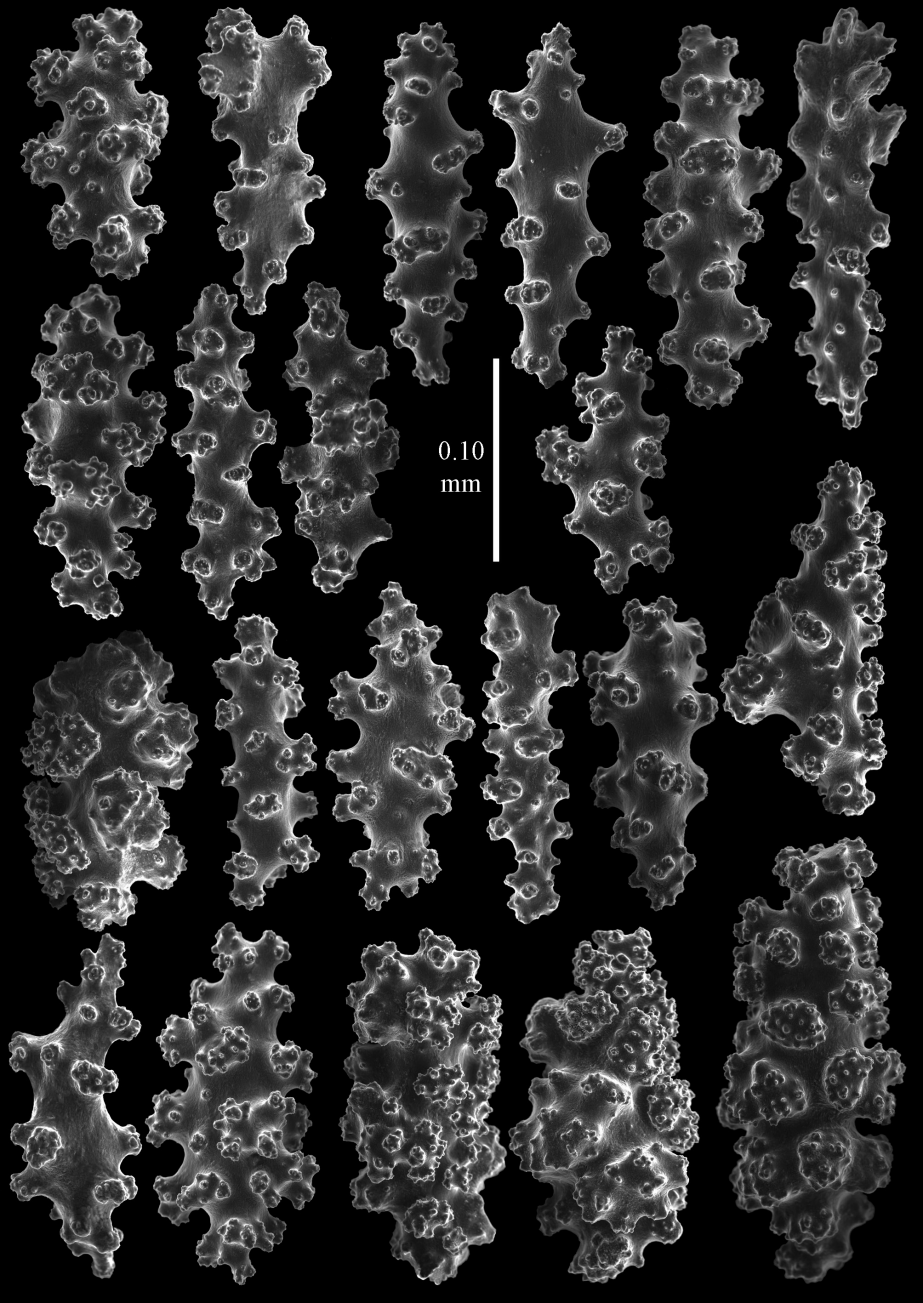
*Euplexaura yayoii* sp. n., holotype RMNH 42104 sclerites from surface layer of coenenchyme.

Figures 1 , 2e , 10 , 11

#### Material examined.

Holotype RMNH 42104 (AKM1551), Off Ohako-zaki cape, Otsuchi Bay, Iwate Prefecture, Japan. 142°00.640'E 39°31.400'N, depth 77.0 m, *RV Yayoi*, st. 4-1, coll. A.K. Matsumoto, 27 April 2010; paratypes RMNH 42105 (AKM592), entrance of Otsuchi Bay, Iwate Prefecture, Japan, 39°21.858'N, 141°59.972'E, depth 65.6 m, *RV Yayoi*, st. 1, coll. A.K. Matsumoto, 12 September 2005; RMNH 42106 (AKM597), same data as AKM 592; RMNH 42107 (AKM623), off Ohako-zaki cape, Otsuchi Bay, Iwate Prefecture, Japan, 142°00.556'E 39°21.452'N, depth 63 m, *RV Yayoi*, st. 2, coll. A.K. Matsumoto, 12 September 2005.

#### Description.

The holotype is 2.5 cm high and 5.5 cm wide (Figure 2e). The colony is branched only once, 1 cm above the base. The two branches are very slender, only 1 mm thick; the calyces are low, dome-shaped, arranged spirally around the branches.

The polyps have points with slightly bent, flattened spindles, up to 0.30 mm long, with a few tubercles and a slightly spiny distal end (Figure 10b). The collaret has bent, flattened spindles, up to 0.30 mm long, with simple tubercles, the largest tubercles present in the middle (Figure 10c). The tentacles have flattened rods, up to 0.15 mm long, with hardly any tubercles (Figure 10a). The surface layer of the branches has spindles and blunt ellipsoids, up to 0.15 mm long, with complex tubercles (Figure 11). The deeper layer has short spindles, up to 0.10 mm long, and a few crosses (Figure 10d-e); all with simple tubercles.

#### Etymology.

Named after the research vessel that was used to collect the specimens.

#### Remarks.

The live colony has blue-coloured polyps. *Euplexaura yayoii* differs from the two other Japanese *Euplexaura* species with thread-like branches, *Euplexaura boninensis* and *Elasmogorgia mitsukurii*, by its very small sclerites.

#### Distribution.

Otsuchi Bay, Iwate Prefecture, Japan.

## Discussion

Originally, there were four species of *Elasmogorgia*: *Elasmogorgia filiformis* Wright & Studer, 1889, *Elasmogorgia filigella* Thomson and Dean 1931, *Elasmogorgia flexilis* Hickson, 1905, and *Elasmogorgia ramosa* Nutting, 1912. Based on the present re-examination, it is obvious that *Elasmogorgia filiformis*, with spindles covered by simple tubercles, is not a species of *Thesea*. Corals of this genus have coarse rugose plates, sometimes tuberculate spindles and double heads (Bayer 1981). Therefore the genus *Elasmogorgia* is reinstated here. The only two species from Japan previously recognized as *Filigella*, *i.e.*, *Filigella mitsukurii* and *Filigella boninensis*, were re-examined and both proved to belong to the genus *Euplexaura*. *Elasmogorgia filigella* from Kalimantan is a species of *Astrogorgia*. This leaves *Elasmogorgia ramosa* and *Elasmogorgia flexilis* unexamined. *Elasmogorgia ramosa* was collected by the Steamer Albatross at Satamisaki Light, south of Kyushu I., Kagoshima prefecture, Japan, 103 fms (188 m), and *Elasmogorgia flexilis* from the Maldives. From the descriptions of these two species it is obvious that *Elasmogorgia ramosa*, with a heavily branched colony, is not a *Thesea* or *Elasmogorgia*. *Elasmogorgia flexilis*, with spindles with complex tubercles probably is a species of *Euplexaura*, and therefore the genus *Elasmogorgia* is considered here monotypic with *Elasmogorgia filiformis* as its only member. *Elasmogorgia filiformis* mostly resembles a species of *Astrogorgia*. Following Bayer (1981) we also consider *Filigella* a synonym of *Thesea*. All Japanese species previously included in *Filigella* are assigned to *Euplexaura* in this study.

All Japanese thread-like plexaurid material South of Kashima Sea was previously identified as *Filigella mitsukurii* and it clearly is the most common thread-like plexaurid species of Japan.

## Supplementary Material

XML Treatment for Elasmogorgia

XML Treatment for Elasmogorgia filiformis

XML Treatment for Euplexaura

XML Treatment for Euplexaura arbuscula

XML Treatment for Euplexaura boninensis

XML Treatment for Euplexaura mitsukurii

XML Treatment for Euplexaura yayoii
